# Predation Limits Spread of *Didemnum vexillum* into Natural Habitats from Refuges on Anthropogenic Structures

**DOI:** 10.1371/journal.pone.0082229

**Published:** 2013-12-12

**Authors:** Barrie M. Forrest, Lauren M. Fletcher, Javier Atalah, Richard F. Piola, Grant A. Hopkins

**Affiliations:** 1 Coastal and Freshwater Group, Cawthron Institute, Nelson, New Zealand; 2 Maritime Division, Defence Science and Technology Organisation, Melbourne, Victoria, Australia; Université du Québec à Rimouski, Canada

## Abstract

Non-indigenous species can dominate fouling assemblages on artificial structures in marine environments; however, the extent to which infected structures act as reservoirs for subsequent spread to natural habitats is poorly understood. *Didemnum vexillum* is one of few colonial ascidian species that is widely reported to be highly invasive in natural ecosystems, but which in New Zealand proliferates only on suspended structures. Experimental work revealed that *D. vexillum* established equally well on suspended artificial and natural substrata, and was able to overgrow suspended settlement plates that were completely covered in other cosmopolitan fouling species. Fragmentation led to a level of *D. vexillum* cover that was significantly greater than was achieved as a result of ambient larval recruitment. The species failed to establish following fragment transplants onto seabed cobbles and into beds of macroalgae. The establishment success of *D. vexillum* was greatest in summer compared with autumn, and on the underside of experimental settlement plates that were suspended off the seabed to avoid benthic predators. Where benthic predation pressure was reduced by caging, *D. vexillum* establishment success was broadly comparable to suspended treatments; by contrast, the species did not establish on the face-up aspect of uncaged plates. This study provides compelling evidence that benthic predation was a key mechanism that prevented *D. vexillum*’s establishment in the cobble habitats of the study region. The widespread occurrence of *D. vexillum* on suspended anthropogenic structures is consistent with evidence for other sessile invertebrates that such habitats provide a refuge from benthic predation. For invasive species generally, anthropogenic structures are likely to be most important as propagule reservoirs for spread to natural habitats in situations where predation and other mechanisms do not limit their subsequent proliferation.

## Introduction

Human activities in the marine environment have led to the creation of extensive areas of artificial habitat (e.g. floating pontoons, wharf piles, aquaculture structures) along coastal margins [1], [2]. As non-indigenous species (NIS) can be dominant fouling organisms in such habitats, the role of anthropogenic structures in facilitating NIS establishment and spread is increasingly being recognized [3]–[6]. An important question that remains poorly understood is the extent to which populations of NIS on artificial structures act as reservoirs for invasion into natural systems [2], [7]. Although the community composition and dominant species inhabiting artificial structures often differs greatly to that on the adjacent natural seabed [7]–[11], there are a number of non-indigenous fouling organisms that are also highly invasive in natural systems [3], [12]–[16]. Among these, the ascidians are a group that often dominate the fouling biomass on suspended artificial structures, but whose invasiveness into natural habitats is highly variable among species, and within species among different geographic regions [7], [17], [18].

The colonial ascidian *Didemnum vexillum* Kott [19] has been widely described as forming highly invasive populations in natural habitats. The most dramatic reported example of invasiveness in this species (hereafter referred to as *Didemnum*) is an extensive population covering 230 km^2^ of pebble and gravel habitat (up to 65 m water depth) on Georges Bank off the northeastern coast of the United States [16], [20]. In the last 10–15 years, non-indigenous populations of *Didemnum* have also been reported from many other regions, including: the west coast of the United States (including southern Alaska), British Columbia (Canada), the United Kingdom, Ireland, northern France, the Netherlands, northern Italy and New Zealand [19], [21]–[26]. Although *Didemnum* is most prevalent on artificial structures in these locations, it has also been described from natural rocky substrata, macroalgal beds, seagrass habitats, tide pools, estuaries, lagoons, and open coastal areas [27]–[29].

In contrast to these examples, New Zealand is one of few regions where *Didemnum* is prolific on artificial structures (including vessel hulls), but appears to have only a limited ability to establish in natural habitats [30]. Where invasion of such habitats occurs in New Zealand, it is evident only in situations where *Didemnum* colonies are not in direct contact with natural seabed substrata. In the Marlborough Sounds region where *Didemnum* is most widespread, the species occurs in natural habitats only where there is biogenic structure such as that provided by horse mussels and macroalgal canopies, or where debris such as submerged logs are present (Table 1). However, such populations are not common, and have only been recorded in localities that have infestations on adjacent marine farms and other structures. In contrast, regional surveys conducted during a *Didemnum* management program ending in 2008, detected populations of *Didemnum* on approximately 123 artificial structures, but did not record the species from rocky (primarily cobble) subtidal habitats that were adjacent.

**Table 1:** Habitats in Marlborough Sounds study region on which *Didemnum* has been recorded or has established.

Habitat	Description
**Artificial structures floating or elevated off seabed**	
Floating structures	Widespread and abundant on mussel farms, salmon farms, floating pontoons and mooring lines
Fixed elevated structures	Widespread and abundant on wharf piles
**Biogenic features and seabed structures or debris**	
Erect sessile epifauna	Present on horse mussels, finger sponges and hydroid trees adjacent to infected artificial structures
Erect macroalgae	Present on canopy-forming macroalgae (*Carpophyllum flexuosum* & *Macrocystis pyrifera*), adjacent to infected artificial structures
Mobile epibiota	Decorator crabs adjacent to infected artificial structures
Organic/inorganic debris	Common on submerged logs, cables and other debris adjacent to infected artificial structures
Artificial rock walls	Abundant in one location on rip-rap beneath heavily infected wharf piles

Previous studies with ascidians (both invasive and indigenous species) have highlighted competition [31]–[33], predation [34]–[38] and the diversity of benthic assemblages [33], [39], as factors that can reduce the establishment and persistence of populations; limited evidence suggests these factors may also be important in the case of *Didemnum* [40], [41]. Predation in particular has the potential to not only limit, but also prevent, some non-indigenous species from establishing in natural habitats. This is in contrast to suspended structures, which may provide a refuge from predation by benthic invertebrates [42]. For example, a study from Chile showed that the non-indigenous solitary ascidian *Ciona intestinalis* was able to establish abundant populations on suspended structures, but could not establish in adjacent natural habitats unless predators were excluded using cages [17].

**Figure 1:**
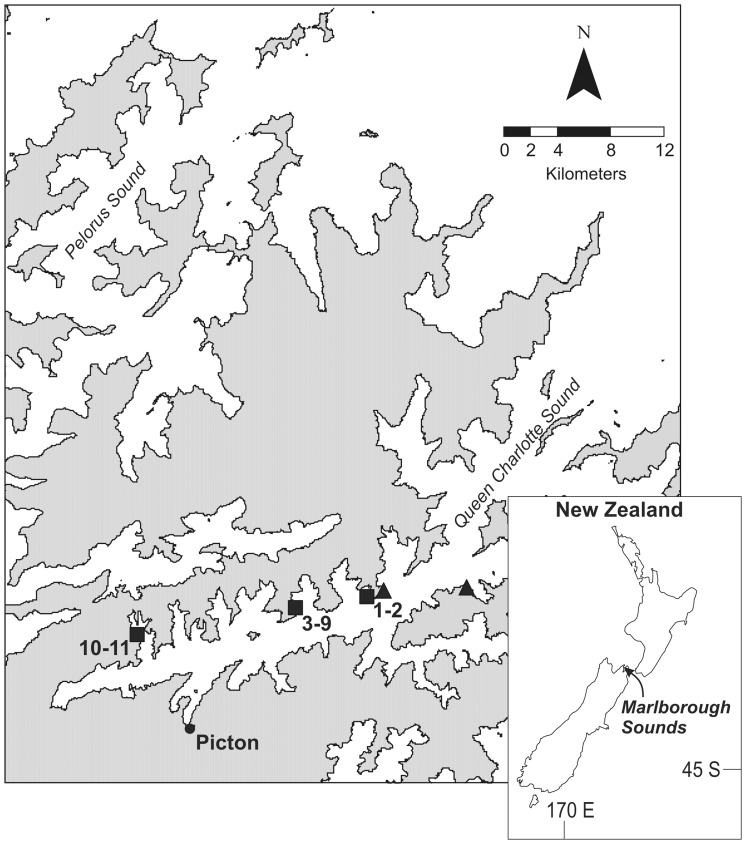
Sites used for *Didemnum vexillum* experiments. Transplants of *Didemnum* fragments were conducted along 11 transects (1–11) across three main locations (filled squares) also used for small scale experiments: Ruakaka Bay (1–2), Blackwood Bay (3–9) and Onahau Bay (10–11). Filled triangles indicate locations of fragment transplants into macroalgal (*Carpophyllum flexuosum*) beds.

The present paper describes a range of experiments designed to examine processes that affect the establishment of *Didemnum* on suspended and rocky subtidal habitats in New Zealand’s Marlborough Sounds (41^o^13′S 174^o^07′E; Fig. 1). Based on experimental evidence, as well as field observations, we consider the potential for *Didemnum* to establish in natural seabed habitats, and the role of reservoir populations on artificial structures in the spread and establishment of the species.

## Materials and Methods

### Ethics Statement and Data Availability

No permits were required for the described study, which complied with all relevant regulations. No locations were privately-owned or protected in any way and the studies did not involve endangered or protected species. All data are available for inspection at the Cawthron Institute.

### Establishment in Relation to Resident Fouling and Substratum Type

*Didemnum* is a colonial species that has the capacity to establish from planktonic larval recruitment and via the reattachment of fragmented colony tissue [43]–[45]. Two initial experiments evaluated the relative importance of recruitment and fragment inoculation, and tested the hypothesis that *Didemnum* could establish equally well on different substratum types (Table 2). The first of these experiments investigated the influence of resident fouling communities. Roughened black Perspex settlement plates (20×20 cm, n = 3 per treatment as described below) were deployed in a horizontal orientation approximately 1 m beneath the sea surface for three months at a location free of *Didemnum*, until their underside had developed a complete two-dimensional fouling layer. These pre-fouled plates, consisting almost exclusively of colonial ascidians (*Botrylloides leachi*, *Botryllus schlosseri*, *Diplosoma listerianum*) and the encrusting bryozoan *Watersipora subtorquata*, were subsequently suspended among an extensive *Didemnum* population on a floating salmon aquaculture cage at Ruakaka Bay (Fig. 1), along with bare plates. Triplicate pre-fouled and bare plates had a small (∼20×40 mm) fragment of *Didemnum* colony attached to them (secured by rubber band), and were randomized among triplicate pre-fouled and bare plates that had no *Didemnum* attached. Hence, whereas all plates were exposed to ambient larval recruitment, half of them were additionally subjected to a single fragment inoculation event. Plates were suspended horizontally facing down (0.5–1.5 m depth) for three months (17 January to 21 April 2008) during the peak summer period of *Didemnum* larval recruitment in the study region [46]. At monthly intervals during the three month period, the plates were photographed and colony percentage cover subsequently estimated using ImageJ software [47]. Any *Didemnum* colonies present after three months were considered to be successfully established, on the assumption that new colonies could have reproduced in that time. Reproductive maturity in *Didemnum* can be reached two weeks after inoculation with fragments [48], and occurs within two months after larval settlement in the case of other colonial ascidians [49], [50].

**Table 2:** Description of experiments conducted with *Didemnum*.

Experimental component	Substratum and position	*Didemnum* inoculation
**A) Effect of resident fouling and substratum**		
Resident fouling	Suspended bare vs pre-fouled settlement plates	Fragments vs ambient larvae
Substratum	Suspended settlement plates vs cobbles	Fragments vs ambient larvae
**B) ** ***Didemnum*** ** transplant to seabed habitats**		
Regional fragment transplant to natural cobbles	Seabed cobbles	Fragments
Fragment transplant to algal beds (*Carpophyllum flexuosum*)	Mid-canopy & canopy base, seabed withincanopy & canopy edge	Fragments
**C) Seabed invasion resistance/caging experiments**		
Blackwood Bay summer 2008	Suspended, seabed caged, seabed uncaged	Fragments vs ambient larvae
Blackwood Bay autumn 2008	Suspended, seabed caged, seabed uncaged	Fragments vs pre-established *Didemnum*
Onahau Bay summer 2009	Suspended, seabed uncaged	Fragments vs ambient larvae

All treatments in components A and C were exposed to ambient larval inoculation from adjacent reproductive *Didemnum* populations. Invasion resistance experiments in component C also included a comparison of face-up and face-down orientations. See Fig. 1 for experimental locations.

In the second experiment (see Table 2), the cover of *Didemnum* developing from ambient larval inoculation and fragments was compared between replicate (n = 4) settlement plates and natural greywacke cobbles of comparable surface area, which were collected from the study site. The selected cobbles were devoid of visible macrofouling, and were centre-drilled so that they could be hung in a similar manner to the plates. Plates and cobbles were suspended horizontally facing down (0.5–1.5 m depth) for three months at Ruakaka Bay (Fig. 1). The cover of *Didemnum* was determined at the end of the three month deployment, and was expressed as surface area (cm^2^) to account for the differences in cobble sizes.

### Transplant of *Didemnum* to Seabed Habitats

Two transplant experiments were conducted in January 2008 (austral summer) to examine the regional-scale establishment potential of *Didemnum* in the Marlborough Sounds (Table 2). Firstly, large colony fragments (∼100×200 mm) were attached to cobbles and transplanted to subtidal cobble habitats along each of 11 transects (1–16 m depth, 10 m long, n = 102 in total) in three main locations: Ruakaka Bay, Blackwood Bay and Onahau Bay (Fig. 1). The transects were revisited over the following 24 hours, and after 1 month, and the presence/absence of *Didemnum* colonies was determined.

Secondly, fragments were transplanted into canopies of a fucoid macroalgal species (*Carpophyllum flexuosum*) that *Didemnum* had previously been observed to establish on (see Table 1). It was hypothesized that the erect biogenic structure provided by the algal canopy would elevate *Didemnum* from the seabed and provide a refuge from predation. Two plots (a treatment and reference plot) of *C. flexuosum* were identified in each of two locations (Fig. 1), each covering an area of at least 10×10 m at approximately 2 m depth. Ten *Didemnum* fragments (∼30×100 mm) were transplanted into the treatment plots in each of four positions: (i) within canopy approximately 1 m from the seabed, (ii) within canopy at the base of the stipe of each *C. flexuosum*, (iii) within canopy on the seabed, and (iv) at the edge of each canopy on the seabed (i.e. at the distinct boundary between the canopy and barren rock habitat). For each of the ten canopy transplants, fragments were attached to ten separate tagged *C. flexuosum* plants. The plots were revisited over the following 24 hours, and after 1 month, and the presence/absence of *Didemnum* colonies was determined.

### Experimental Evaluation of Seabed Invasion Resistance

A series of field caging experiments was undertaken to test the hypothesis that *Didemnum* could establish on (or survive transplant to) the seabed, if benthic predation was reduced (Table 2). The overall experimental design compared establishment success among settlement plates (20×20 cm) that were deployed in two horizontal orientations (face-up and face-down), and which were either: (i) uncaged on the seabed, (ii) fully caged on the seabed using a 10 mm stainless mesh screen (to exclude larger predators), or (iii) suspended at comparable depths from adjacent suspended structures that were infected with *Didemnum*. Seabed cages and plates were attached to gridded (150×150 mm) steel reinforcing mesh. Previous studies in the region have shown that the cage design and method does not introduce experimental artefacts [44].

Caging experiments were first conducted for three months (17 January –11 April 2008) during the recruitment peak at the study site in Blackwood Bay (Fig. 1). Seabed caged and uncaged plates were deployed at approximately 10 m depth, and arranged 25 m distance from a floating pontoon (dimensions ∼2×4 m) that had been pre-seeded with approximately 350 kg of reproductive *Didemnum* [51]. Uncaged plates were also suspended beneath the pontoon at a similar depth. A fragment of *Didemnum* was attached to one set of plates (n = 6) and colony percentage cover measured after three months. The ambient larval supply from the pontoon was simultaneously measured on an additional set of plates (n = 3 suspended, n = 6 seabed caged and uncaged) that were deployed for the same period, and involved counting the density of recruits in the laboratory using a binocular microscope. Daily mean water temperature during the experiment were determined from hourly measurements with a TidbiT v2 Temperature Logger, and ranged from 17.0 to 19.6°C.

The fragment inoculation component of the caging experiment was repeated during April to June 2008, covering a two month autumn to mid-winter period in New Zealand. Daily mean water temperatures of 13.1 to 17.1°C during the second experimental period were within the species optimal range [25], [29], and *Didemnum* was still actively recruiting throughout this period. Simultaneous with the fragment inoculation experiment, the persistence of pre-established *Didemnum* colonies was assessed using the same experimental design (n = 6 per treatment). For this component, the colonies were pre-established on settlement plates using fragments. The resultant colony cover on the plates at the beginning of the experiment ranged from 50 to 92% (mean 72.1% ±1.7SE) and plates were randomly allocated across treatments. Results were expressed as colony survival, reflected as the change in percentage cover at the completion of the experiment.

The fragment inoculation and recruitment experiments conducted in summer 2008 were repeated at a different location (Onahau Bay, see Fig. 1) in summer 2009, with the exception that the cage treatment was not included (the suspended versus seabed contrast was of primary interest) (see Table 2). The infected structure at the second location was a suspended pontoon of similar size to that in Blackwood Bay; however, site constraints meant that seabed plates were deployed on shallow cobbles (2–3 m depth) immediately adjacent to the pontoon. *Didemnum* percentage cover (from fragments) and recruit density on suspended and seabed plates (n = 4) was assessed after three months, as described above.

### Statistical Analyses

A two-way repeated-measures analysis of variance (RM-ANOVA) was used to test for differences through time in the percentage cover of *Didemnum* on bare and pre-fouled plates at the Ruakaka site. The experimental design included ‘Inoculation’ (two levels: Fragments and Recruits) and ‘Fouling’ (two levels: Bare and Pre-fouled) as fixed orthogonal factors. Data from the substratum type experiment were analyzed using a two-way ANOVA with ‘Inoculation’ (two levels: Fragments and Recruits) and ‘Substratum’ (two levels: ‘Plate’ and ‘Cobble’) as fixed orthogonal factors.

The Blackwood Bay caging experiments involved two-way ANOVA analyses with ‘Treatment’ (three levels: Cage, No Cage, Suspended) and ‘Orientation’ (two levels: face-up, face-down) as fixed orthogonal factors. Data from the summer and autumn experiments resulted in a total of three response variables (recruit density, colony cover and colony survival), which were analyzed separately in the same manner. For the summer 2008 recruit density data, one of the ‘Suspended-Down’ and ‘Suspended-Up’ replicates was lost during the experimental phase, so the analysis for this experiment was consequently unbalanced. Recruit density and colony cover data from the Onahau Bay experiments were analyzed separately using the same design, with the exception that there were only two treatment levels: ‘Suspended’ and ‘No Cage’.

Due to data over-dispersion and a high proportion of zero values in some treatments, all ANOVA analyses were performed using a permutational approach [52], with the PERMANOVA add-in of PRIMER 6 software [53]. Analyses used resemblance matrices based on Euclidean distance, and the partial sum of squares (Type III) with 9999 permutations of residuals under a reduced model. Data were log (x+1) transformed to achieve approximate unimodal symmetry and to avoid right skewness. Significant terms were then investigated using *a posteriori* pair-wise comparisons with the PERMANOVA *t*-statistic and 999 permutations.

## Results

### Establishment in Relation to Resident Fouling and Substratum Type

**Figure 2:**
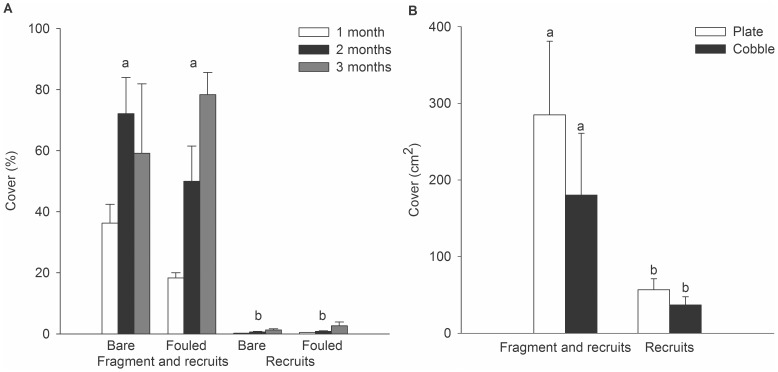
Establishment of *Didemnum* on different substrata. In two separate experiments of three months duration, different substrata were exposed to ambient larval recruitment, with half of them also inoculated with a *Didemnum* fragment. A) Mean *Didemnum* cover (% cover ±1SE) on bare settlement plates compared with plates that were pre-fouled with other sessile species. B). Mean *Didemnum* cover (cm^2^±1SE) on bare settlement plates compared with cobbles that were collected from the adjacent intertidal zone. In both experiments, substrata were suspended between 0.5 and 1.5 m deep. Different letters (a,b) indicate significant differences as indicated by the PERMANOVA post-hoc *t*-test.

*Didemnum* established equally well on settlement plates, irrespective of whether they were bare or pre- fouled; however, inoculation with fragments led to a colony cover that was considerably greater than that arising from ambient recruitment alone (Fig. 2A). On pre-fouled plates, microscopical observation revealed new recruitment and colony growth on top of the existing fouling. After three months, plates inoculated with fragments had a mean colony cover of 59.2 to 78.3% (±23.7 and 7.3%, respectively) compared with <3% cover on plates exposed to larval recruitment alone. Hence, there was a statistically significant effect of inoculation type, which was consistent over time (Table 3A). In contrast, there was no overall effect of substratum, but the substratum effect was variable over time (Table 3A, Substratum×Month, p<0.05). Even after one month, the *Didemnum* cover resulting from fragment inoculation (mean 36.3±6.1 and 18.3±1.6% for bare and pre-fouled plates, respectively) was already considerably greater than achieved from ambient recruitment. The comparison of plates and cobbles yielded a similar pattern, with significantly greater colony cover arising from fragment inoculation compared to larval recruitment, but no significant effect of substratum type (Fig. 2B, Table 3B).

**Table 3:** Effects of inoculation and substratum on *Didemnum* cover.

Source	df	MS	F	Sig	
**A) Bare and pre-fouled plates**					
Inoculation	1	92.60	397.9	**	
Substratum	1	0.00	0.0		
Inoculation×Substratum	1	0.39	1.7		
Month	2	1.95	28.3	***	
Inoculation×Month	2	0.24	3.4		
Substratum×Month	2	0.35	5.1	*	
Inoculation×Substratum×Month	2	0.17	2.5		
Residual	16	0.07			
**B) Plates and cobbles**					
Inoculation	1	7.83	13.28	**	
Substratum	1	1.33	2.25		
Inoculation×Substratum	1	0.05	0.09		
Residual	12	0.59			

A) RM-ANOVA comparing percentage cover from fragment and larval inoculation on bare and pre-fouled plates; B) Permutational ANOVA comparing percentage cover from fragment and larval inoculation on plates and cobbles. Statistical significance denoted as follows: * = p<0.05, ** = p<0.01, *** = p<0.001.

### Transplant of *Didemnum* to Seabed Habitats

**Figure 3:**
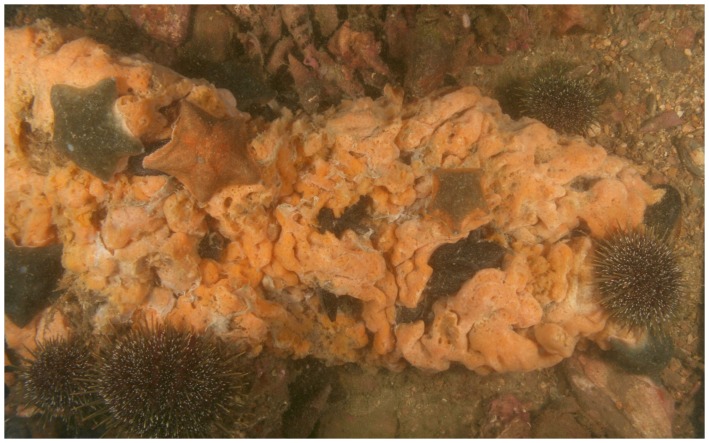
Predation on *Didemnum* following transplant to the seabed. Image shows predation by cushion stars, *Patiriella regularis*, and sea urchins, *Evechinus chloroticus*, on a large (∼20×35 cm) fragment of *Didemnum*. Predation by these and/or other species was observed following fragment inoculation into beds of the macroalga *Carpophyllum flexuosum*, and when settlement plates with fragments or pre-established *Didemnum* colonies were placed on the seabed.

The regional-scale transplant of large *Didemnum* fragments along 11 transects in cobble habitats failed to lead to any colonies establishing. In fact, within hours of transplant, fragments were usually covered by benthic predators, which subsequently consumed intact colony tissue (i.e. not just cut edges). Most prevalent among the predators observed to actively consume the fragments were small (up to 60 mm diameter) cushion stars, *Patiriella regularis*, and sea urchins, *Evechinus chloroticus* (Fig. 3). However, large eleven-arm sea stars (*Coscinasterias muricata*), top shells (*Turbo smaragdus*), chitons (*Cryptoconchus porosus*) and hermit crabs (Paguridae) were also observed on the fragments and were presumed to be eating them.

*Didemnum* also failed to establish in the *Carpophyllum flexuosum* transplant experiment. In the case of the seabed transplants (adjacent to and at the edge of the *C. flexuosum* canopy), this result was consistent with the regional-scale transplant experiment, with predation on the fragments observed within the first day of the experiment. However, *Didemnum*’s failure to establish following transplant to the basal stipe of *C. flexuosum*, and especially the canopy, was contrary to our expectations. The seastar *C. muricata* was observed to consume one of the basal transplants, and one of the canopy transplants had *T. smaragdus* on it. One month later, neither the transplanted fragments nor their remains were visible.

### Seabed Invasion Resistance

**Figure 4:**
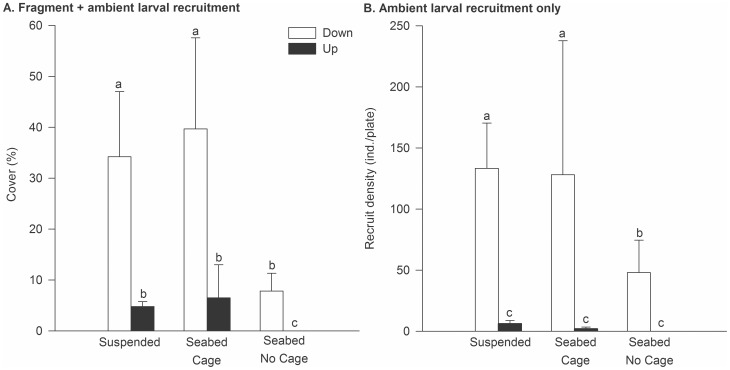
Establishment of *Didemnum* in suspended and seabed treatments in summer 2008 at Blackwood Bay. Perspex plates were positioned in face-up and face-down orientations, with the seabed treatment including a Cage and No Cage comparison. All plates were exposed to larval recruitment from a floating pontoon approximately 25 m away, for a three month experimental duration. A). Mean *Didemnum* cover (% cover ±1SE) on plates inoculated with *Didemnum* fragments. B). Mean density of *Didemnum* recruits (±1SE) on plates subjected only to ambient larval supply. Different letters (a,b,c) indicate significant differences as indicated by the PERMANOVA post-hoc *t*-test.

During the summer of 2008 in Blackwood Bay, *Didemnum* established a greater colony cover and recruited at greater densities on face-down plates compared to face-up ones (Orientation, p<0.01, Table 4A, Figure 4A). Additionally, there was a significant Treatment effect (Table 4A, Figure 4A), with Suspended plates having greater cover than the seabed No Cage treatment (PERMANOVA, pair-wise comparison p<0.05), but not the seabed Cage treatment. On face-down plates, fragment inoculation led to 34.2 (±12.8) and 39.8% (±17.9) mean cover in Suspended and Cage treatments, respectively, compared with 8% cover (±3.4) on No Cage plates. On face-up plates, *Didemnum* failed to establish without caging, and cover was relatively low but comparable on suspended and caged plates. Patterns in recruitment density largely mimicked the fragment inoculation patterns, with a significant effect of Orientation and Treatment (Table 4B, Figure 4B). No recruits were recorded on the face-up plates unless they were caged or suspended (Fig. 4B).

**Table 4:** Effects on colony percentage cover and recruit density at Blackwood Bay in summer 2008.

Source	df	MS	F	Sig
**A) Cover**				
Treatment	2	7.5	6.1	*
Orientation	1	27.7	22.3	**
Treatment×Orientation	2	0.2	0.2	
Residual	28	1.2		
**B) Recruit density**				
Treatment	2	7.0	4.5	*
Orientation	1	52.2	33.5	**
Treatment×Orientation	2	0.2	0.2	
Residual	24	1.6		

Results show effects of treatment (Suspended, Cage, No Cage) and orientation, from permutational ANOVA. Statistical significance denoted as follows: * = p<0.05, ** = p<0.01, *** = p<0.001.

**Figure 5:**
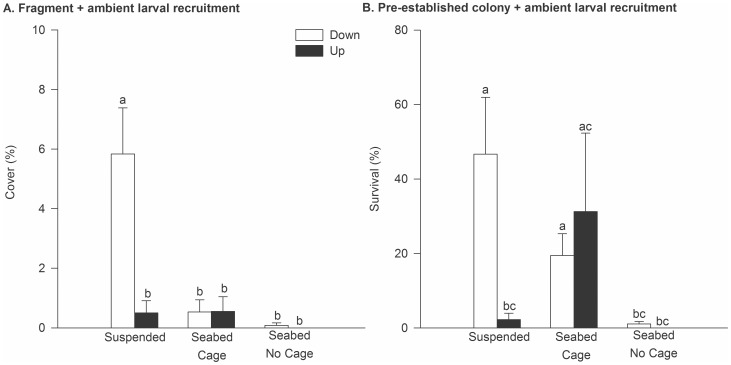
Establishment of *Didemnum* in suspended and seabed treatments in autumn 2008 at Blackwood Bay. Settlement plates were positioned in face-up and face-down orientations, with the seabed treatment including a Cage and No Cage comparison. All plates were exposed to an ambient larval supply from a suspended pontoon approximately 25 m away, for a two month experimental duration. A). Mean *Didemnum* cover (% cover ±1SE) on plates inoculated with *Didemnum* fragments. B). Mean survival of *Didemnum* (% survival ±1SE) colonies on plates for which a colony cover (mean ∼73%) was pre-established at the start of the experiment. Different letters (a,b,c) indicate significant differences as indicated by the PERMANOVA post-hoc *t*-test.

In autumn 2008 at Blackwood Bay, *Didemnum* establishment by fragments was poor compared with the previous summer (Fig. 5A), but was consistent with the fact that the persistence of pre-established colonies was simultaneously low (Fig. 5B). In the fragment inoculation experiment, a significant Treatment×Orientation interaction (Table 5A) reflected the significantly greater *Didemnum* cover on face-down suspended plates (6% cover) than in all other treatments in which cover was <1% (Fig. 5A, PERMANOVA, pair-wise comparison p<0.05). On No Cage plates, no *Didemnum* was recorded in the face-up orientation (Fig. 5A), nor did any pre-established *Didemnum* persist in that orientation (Fig. 5B). By contrast, survival was significantly greater in seabed Cage and face-down Suspended treatments (Fig. 5B, PERMANOVA, pair-wise comparison p<0.05). Whereas mean survival was comparable for the two orientations in the Cage treatments (19.5±5.8 and 31.2±21.1% for face-down and face-up plates, respectively), the face-up Suspended plates became covered in a layer of filamentous algae and *Didemnum* survival was poor (Fig. 5B).

**Table 5:** Effects on colony percentage cover and survival at Blackwood Bay in autumn 2008.

Source	df	MS	F	Sig
**A) Cover**				
Treatment	2	3.1	14.6	
Orientation	1	2.5	11.5	
Treatment×Orientation	2	2.1	9.7	***
Residual	30	0.2		
**B) Survival**				
Treatment	2	14.8	10.6	
Orientation	1	13.4	9.6	
Treatment×Orientation	2	5.3	3.8	*
Residual	30	1.4		

Results show effects of treatment (Suspended, Cage, No Cage) and orientation, from permutational ANOVA. Statistical significance denoted as follows: * = p<0.05, ** = p<0.01, *** = p<0.001.

**Figure 6:**
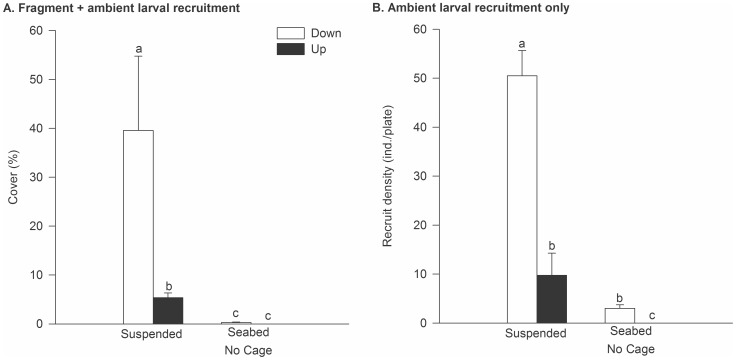
Establishment of *Didemnum* in suspended and seabed treatments in summer 2009 at Onahau Bay. Settlement plates were positioned in face-up and face-down orientations, with suspended plates compared to uncaged seabed plates. All plates were exposed to an ambient larval supply from a suspended pontoon 2–3 m away, for a three month experimental duration. A). Mean *Didemnum* cover (% cover ±1SE) on plates inoculated with *Didemnum* fragments. B). Mean density of *Didemnum* recruits (±1SE) on plates subjected only to ambient larval supply. Different letters (a,b,c) indicate significant differences as indicated by the PERMANOVA post-hoc *t*-test.

The experiment conducted in summer 2009 at Onahau Bay generally confirmed the importance of orientation, and the greater establishment (Fig. 6A) and recruitment (Fig. 6B) of *Didemnum* in suspended compared with seabed habitats. In the fragment inoculation experiment, a significant Treatment×Orientation interaction (Table 6A) indicated an orientation effect for Suspended, but not Seabed plates (see Fig. 6A, PERMANOVA, pair-wise comparison p<0.05). A similar general pattern was evident for larval recruitment (Fig. 6B), although significant effects of both Treatment and Orientation were detected (Table 6B).

**Table 6:** Effects on colony percentage cover and recruit density at Onahau Bay in summer 2009.

Source	df		MS	F				Sig
**A) Cover**								
Treatment	1		25.7	146.0				
Orientation	1		3.7	20.8				
Treatment×Orientation	1		2.1	11.9				**
Residual	12		0.2					
**B) Recruit density**								
Treatment	1		21.7	89.3				***
Orientation	1		10.2	41.7				***
Treatment×Orientation	1		0.2	1.0				
Residual	12		0.2					

Results show effects of treatment (Suspended, No Cage) and orientation, from permutational ANOVA. Statistical significance denoted as follows: * = p<0.05, ** = p<0.01, *** = p<0.001.

## Discussion

### Invasiveness of *Didemnum*

Substratum type and the presence of an established fouling assemblage were unimportant as explanatory variables for the invasion success of *Didemnum*. The species established equally well on settlement plates and cobbles, and was able to recruit to, and subsequently overgrow, cosmopolitan fouling taxa such as colonial ascidians and encrusting bryozoans. The latter finding is consistent with field observations and manipulative experiments from a number of localities world-wide, which indicate the ability of *Didemnum* and other ascidians to overgrow other sessile organisms [16], [18], [54], [55]. A single exception for *Didemnum* is evident from a recent experimental study (∼10 weeks duration) in Long Island Sound, which suggested that invasion success was linked to the availability of unoccupied space [41].

*Didemnum* revealed a greater ability to establish as a result of fragment inoculation than ambient larval recruitment. This was evident in experiments that compared different substrata (see Fig. 2), and was also observed in the field during the Blackwood and Onahau Bay experiments. In fact, at Blackwood Bay, the cover of *Didemnum* resulting from ambient recruitment was not measurable by image analysis. The important role of fragments in the establishment of *Didemnum* [43] and other colonial ascidians [56] has previously been recognized. Natural fragmentation in the species arises from the tendril-like lobes that colonies often exhibit [57], and fragments can remain viable for as long as four weeks when artificially kept in suspension [45]. Although *Didemnum* can reattach from fragments as small as 5×3 mm, survivorship increases with fragment size under field conditions [44]; thus, relatively large fragments were used in the present study to maximize the likelihood that they would be viable and able to reattach.

The non-measurable cover resulting from recruitment at Blackwood Bay is perhaps explained by the fact that the experimental plates were up to 25 m from the *Didemnum* larval source, whereas plates were only 1–3 m from the larval source in the other experiments, and less subjected to dilution processes. This hypothesis is consistent with parallel research in the study region that described an exponential decline in *Didemnum* recruitment within tens of meters from reproductive populations [51], and also consistent with the notion that invasion success can depend on ‘propagule pressure’ [58], [59]. However, a propagule pressure hypothesis does not explain why successful recruits (or divided zooids) did not continue to grow at Blackwood Bay, as the three month experimental duration was more than sufficient for this to occur [60], [61]. Competition with other recruiting fouling species seems an unlikely explanation, as the diversity and prevalence of other sessile species at Blackwood Bay was quite low (e.g. consisted of occasional small barnacles, spirorbid and serpulid polychaetes, hydroids, and encrusting bryozoans), and comparable to the other locations. Further investigation is needed to ascertain the extent to which site-specific factors (e.g. environmental conditions, post-settlement processes) may have contributed to differences in recruitment and subsequent colony establishment success among locations.

While the results suggests a more important role for fragmentation in the initial stages of establishment, localized ambient larval recruitment may lead to a comparable level of establishment to fragments in the longer-term (>12 months) [54]. Thus, the apparent advantage in establishment by fragmentation may diminish over time. The findings highlight that despite the potential for dispersal in this species over scales of hundreds of meters and perhaps a few kilometers [51], low level larval recruitment at the outer limits of the dispersal range may not immediately lead to the development of extensive new populations of *Didemnum*, even on suspended artificial structures which generally appear optimal for establishment.

The decline in establishment success of fragments in the autumn experiment at Blackwood Bay (compared with summer) was accompanied by poor survival of pre-established colonies at the time of the mid-winter cessation of the experiment. Such findings are consistent with a seasonal decline in success due to decreasing water temperatures [61], as opposed to a failure of the inoculation method. Thus, even though *Didemnum* can persist and still recruit until mid-winter in the study region [46], the period of greatest risk for establishment and proliferation is clearly during warmer summer months. Despite an apparent seasonal decline in invasion success in cooler months, the fact that *Didemnum* still persists means that established populations will provide a reservoir from which extensive colonies may develop the following summer.

### Invasion Resistance in Natural Habitats

The combination of regional scale transplants, smaller spatial scale experiments, and associated field observations, provided compelling evidence that benthic predation is a key mechanism that limits *Didemnum*’s establishment in the natural cobble habitats of the study region. Simultaneously, the widespread occurrence of the species in habitats that are elevated off the seabed (see Table 1), is consistent with evidence for other sessile marine invertebrates that both anthropogenic and biogenic structures can provide a refuge from benthic predation [17], [37], [62], [63]. However, most studies of *Didemnum* and other colonial ascidians have shown that predation acts to reduce but not prevent establishment in natural habitats [36]–[38], [40]. By contrast, the present study indicates that benthic predation may completely exclude *Didemnum* from certain habitat types. This result is consistent with recent analysis showing that, unlike other didemnid ascidians that contain compounds that are thought to reduce predation [64], *Didemnum* does not contain potent secondary metabolites [26].

In the caging experiments, the establishment success of *Didemnum* was greatest on suspended plates, from which benthic predators would have been completely excluded. Where predation pressure was reduced by caging, *Didemnum* fragment establishment and recruitment yielded broadly similar patterns to suspended treatments. Greater recruitment to the face-down aspect of plates was consistent with the negative phototaxis exhibited by ascidian larvae immediately prior to settlement [61], [65]. Marked variability in establishment success in some caged treatments was evident (see Fig. 4), which possibly reflected incursion into some cages of small predators (in particular juvenile cushion stars, *Patiriella regularis*) that were not excluded by the 10 mm mesh. The low level recruitment and colony cover on uncaged face-down seabed plates was not anticipated, but likely reflects reduced predation pressure. The face-down orientation would have restricted predator access into the spaces among the cobbles, especially in the case of larger-bodied urchins and cushion stars.

The consistent failure of *Didemnum* to establish in any of the seabed transplants, or on the face-up orientation of the No Cage treatments, suggests that predators had relatively unlimited access. In contrast, colonies on face-up plates within cages persisted to a similar extent to that observed on suspended treatments, thus illustrating the invasion potential of *Didemnum* in the absence of benthic predation pressure. Such results are of interest, as a face-up orientation approximates a common scenario in which *Didemnum* forms high-biomass colonies on suspended structures, which can slough off and provide a considerable supply of fragments to the adjacent seabed [30]. However, irrespective of whether *Didemnum* propagules colonize suspended structures or the seabed, subsequent establishment and invasiveness on face-up surfaces may be less than face-down, and subject to a greater suite of limiting factors than predation alone, such as over-settlement by seasonal algae (this study) and smothering by sediment [44].

In addition to these limiting factors, considerable small-scale variability was often evident within the same treatment (e.g. among plates in the same treatment separated by only tens of centimeters), which was not always explainable by extrinsic factors such as differential predation pressure. This variability mirrors that previously observed across small spatial scales in the recruitment and establishment of *Didemnum* and other ascidians [18], [66]. Such findings suggest a stochastic element to successful establishment that may in part be driven by inherent variability in *Didemnum* (e.g. differential viability of fragments from the same or different colonies).

### Propagule Pressure and Invasion Potential of *Didemnum*

Given the apparent stochastic nature of establishment, and the design of our experiments (e.g. short-term with one-off inoculation by fragments) a pertinent question raised by this study is whether increased propagule pressure, such as that provided by a sustained larval and fragment release from an infected anthropogenic structure, could overcome invasion resistance and enhance *Didemnum*’s invasiveness in adjacent natural habitats? This phenomenon has been described theoretically [67], [68], and in empirical studies from a range of aquatic and terrestrial systems that highlight the important interaction between propagule supply and factors that alter mechanisms of invasion resistance [17], [69]–[72].

In the study area, it is apparent that propagule supply alone may not overwhelm invasion resistance, as rocky habitats devoid of *Didemnum* lie adjacent to numerous heavily infected structures throughout the region. However, there is a single location where *Didemnum* has extensively colonized a large (∼10,000 m^2^) area of artificial rip-rap wall (comprising quarry rock) beneath heavily infected piles at an international shipping wharf (see Table 1), yet the invasion has not spread to natural rocky habitats that are contiguous with the rip-rap. A feature of the rip-rap compared with adjacent natural areas is its construction from relatively large angular rocks with many spaces among them, which appeared impoverished in terms of predators and other mobile benthic species (BF, pers. obs.). The reasons are unknown, but the topography of the rip-rap (especially the size of the rocks) may not provide a favorable habitat for mobile species to access or move across. Hence, in this instance, it is conceivably reduced invasion resistance, rather than propagule pressure, that has enabled *Didemnum* to flourish. This hypothesis is consistent with the ‘Persistent Pressure Scenario’ conceptualized in a recent study of the solitary ascidian *Ciona intestinalis* [17]. That scenario proposed that despite a continuous propagule supply from a fouled suspended structure, the establishment of certain marine benthic species may be prevented by predation, unless predation pressure was reduced by processes such as disturbance.

In the present study, predation pressure on *Didemnum* was attributable to a range of species. As these are common species around the New Zealand coastline [73], it may be the case that predation explains the apparent absence of *Didemnum* from rocky seabed habitats elsewhere in the country, despite its increasing prevalence on artificial structures. In general, while anthropogenic structures may function as significant propagule reservoirs that facilitate the spread of invasive species into adjacent natural habitats, proliferation in natural systems may only occur in situations where predation and other mechanisms of invasion resistance are not limiting. That being so, the risk to natural habitats may be exacerbated in situations where a high connectivity of structures (e.g. by natural dispersal) enables them to function as ‘stepping-stones’ for NIS spread [1], [5], [74]. Such spread will increase the number of propagule reservoirs, possibly leading to increased likelihood that invadable natural habitats will eventually be encountered. In order to better understand and effectively manage such risks, there is a need for a greater understanding of the interactions between propagule supply and invasion resistance that preclude or enhance the establishment of invasive species.
